# A Review of Efficacy and Safety of Checkpoint Inhibitor for the Treatment of Acute Myeloid Leukemia

**DOI:** 10.3389/fphar.2019.00609

**Published:** 2019-06-06

**Authors:** Dan Liao, Mengyao Wang, Yi Liao, Jun Li, Ting Niu

**Affiliations:** ^1^Research Laboratory of Hematology, Department of Hematology, West China Hospital, Sichuan University, Chengdu, China; ^2^State Key Laboratory of Biotherapy and Cancer Center, West China Hospital, Sichuan University and Collaborative Innovation Center, Chengdu, China

**Keywords:** checkpoint inhibitor, acute myeloid leukemia, safety, efficacy, immunotherapy

## Abstract

Immune checkpoint inhibitors (ICIs) as positive modulators of immune response have revolutionized the treatment of cancer and have achieved impressive efficacy in melanoma and numerous solid tumor malignancies. These agents are being investigated in acute myeloid leukemia (AML) to further enhance response rate as induction therapy and to improve relapse-free survival (RFS) post chemotherapy and bone marrow transplantation. PD-1 and CTLA-4 are the two most actively investigated checkpoint receptors, which play a role in different stages of anti-tumor immune response. This study reviews data from ongoing phase I, II clinical trials evaluating PD-1 and CTLA-4 inhibitors on AML patients and discusses especially efficacy and adverse events as well as prospects of these drugs in treating AML. Single anti-PD-1 monoclonal antibody infusion shows rather modest clinical efficacy. While combinations of PD-1 inhibitor with hypomethylating agents (HMAs) represent encouraging outcome for relapsed/refractory (R/R) AML patients as well as for elderly patients as first-line therapy option. Adding PD-1 inhibitor to traditional induction therapy regimen is also safe and feasible. CTLA-4 inhibitor ipilimumab exhibits specific potency in treating relapsed AML patients with extramedullary disease in later post-transplantation stage. In terms of side effects, irAEs found in these trials can mostly be appropriately managed with steroids but are occasionally fatal. More rationally designed combinational therapies are under investigation in ongoing clinical trials and will further advance our understanding of checkpoint inhibitors as well as lead us to the most appropriate application of these agents.

## Introduction

Acute myeloid leukemia (AML) is a form of cancer originated from malignant clonal stem cells in bone marrow marked by heterogenous clinical outcome due to the complexity of its molecular and cytogenetic architecture (Dohner et al., 2015). For a long period of time, the treatment options for AML are limited to chemotherapy and hematopoietic stem cell transplantation. However, despite the progression in remission rate with many newly approved chemo-drugs, there are still a bunch of problems that need to be solved regarding treatment efficacy of AML, such as resistance to chemotherapy, relapse after transplantation, and non-tolerance of older patients to high-intensity chemotherapy. Thus, there is a desperate need for innovative approaches. In recent years, with the deepened understanding of the role of immune evasion in tumor maintenance as well as development of immunotherapy, the great wave of antibody therapy is refactoring the field of cancer treatment. Among various immunotherapy approaches, using checkpoint inhibitors to block inhibitory molecules on T cell surface thus reversing T cell from ”exhausted” state to “activated” state to kill tumor cells has proven to be a promising option. Following the success of immune checkpoint inhibitors (ICIs) in solid tumors such as melanoma and non-small cell lung cancer, these drugs are being explored in hematopoietic malignancies including AML (Hodi et al., 2010; O’Day et al., 2010; Rizvi et al., 2015). The inhibition of CTLA-4 and PD-1 are the two most commonly used clinical strategies as immune checkpoint blockade. As proven by the efficacy of allogeneic hematopoietic stem cell transplantation (allo-SCT), leukemia is the typical immune responsive tumor type. Besides, leukemia cells express high level of checkpoint inhibitor receptors for sharing an immune cell lineage, making them potential targets for this treatment (Vollmer et al., 2003; Whiteway et al., 2003; Graf et al., 2005).

## Immunity and Tumor/Acute Myeloid Leukemia

The immune system helps to defend the body against foreign invaders such as bacteria and tumor cells by distinguish between self and non-self. This complex while delicate system plays an essential role in anti-tumor response. Under normal physiological conditions, immune system could recognize a wide variety of neo-antigens expressed on the surface of tumor cells caused by genetic abnormalities (Desrichard et al., 2016). Aside from fusion proteins and mutated proteins, immune system can also recognize the products of non-mutated genes that are preferentially expressed by tumor cells. The effective anti-tumor response contains three main steps (Mellman et al., 2011). Firstly, antigen presenting cells (APCs) such as dendritic cells ingest the antigens, fragment them into antigen peptides, and display them on the surface of the cell joined together with major histocompatibility complex (MHC) molecules. Next, these APCs roam to lymphoid tissues where T cell resides. By recognizing specific peptide-MHC complex, accompanied by costimulatory signals, T cells are activated into effector T cells, which mainly are CD8 positive subpopulation that are capable of attacking infected cells or tumor cells. Finally, the tumor-immune response happens when activated effector T cells infiltrate the tumor bed.

Activating the immune system either passively or spontaneously has long been a goal in cancer treatment for therapeutic benefit. Extraordinary effort has been made throughout history in cancer immunotherapy. On the one hand, doctors fed the patients with anticancer monoclonal antibodies or clear the leukemia cells by the graft-versus-leukemia (GVL) effect when patients receive allogeneic bone marrow transplantation (Ruggeri et al., 2002; Dougan and Dranoff, 2009). These were potent measures for a variety of hematological malignancies as well as solid tumors. On the other hand, scientists tried to provoke spontaneous anti-tumor immunity. Coley, the so-called “father of immunotherapy,” tried to treat his patient with “Coley’s toxins”—the two dead bacteria, *Streptococcus pyogenes* and *Serratia marcescens*—by causing inflammation and destroying tumor cells through activated antibacterial cells. Though this formula remained controversial in the medical field due to the infection risk, Coley’s work showed the possibilities of immunotherapy in cancer, thus leading cancer treatment into a new era (Nossal, 1993).

Among various methods of cancer immunotherapy, inhibiting the immune suppression that contributes a large part to sustaining tumor is of great concern. Cancer cells escape from attacks from immune system by a variety of mechanisms that influence different stages of cancer-immune response circuit. By releasing several kinds of mediators, adenosine for instance, tumors could suppress T-cell activation and enable expansion of regulatory T cells (Treg cells) whose function is to oppose the activity of effector T cells (Ohta, 2016). Another mechanism of tumor to prevent T-cell activation is related to the co-stimulatory signals. Cancer cells with high expression of CTLA4 negatively modulate activated T cells through competitively binding to co-stimulatory molecules on T cell surface (Walunas et al., 1994). Tumor cells can also downregulate their MHC molecule expression to avoid T cell recognition. Up-regulation of several inhibitory molecules such as PD-1 on the surface of tumor cells could cause T-cell anergy or exhaustion after engagement of their ligands on T cells. Based on above mechanisms, several kinds of targeted immunotherapies are under testing, including monoclonal antibodies, immune adjuvants, cytokines, and ICIs. To achieve deeper remission in AML patients, bone marrow transplantation is an effective treatment. Despite the high response rate in some patients, there are still a group of them suffering from disease relapse after transplantation. Studies found that patients with graft-versus-host disease (GVHD) were 2.5 times less likely to relapse compared with those without (Weiden et al., 1979). Lower relapse rate was observed in patients without GVHD who received allografts than those who received identical twin transplants. These results supported an anti-leukemia effect of allogeneic grafts independent of GVHD and suggested the possibility and rationality of boosting immune system to treat AML (Horowitz et al., 1990).

## Checkpoint Inhibition in Acute Myeloid Leukemia: Preclinical Evidences

### Blockade of PD-1 in Acute Myeloid Leukemia

**Figure 1:**
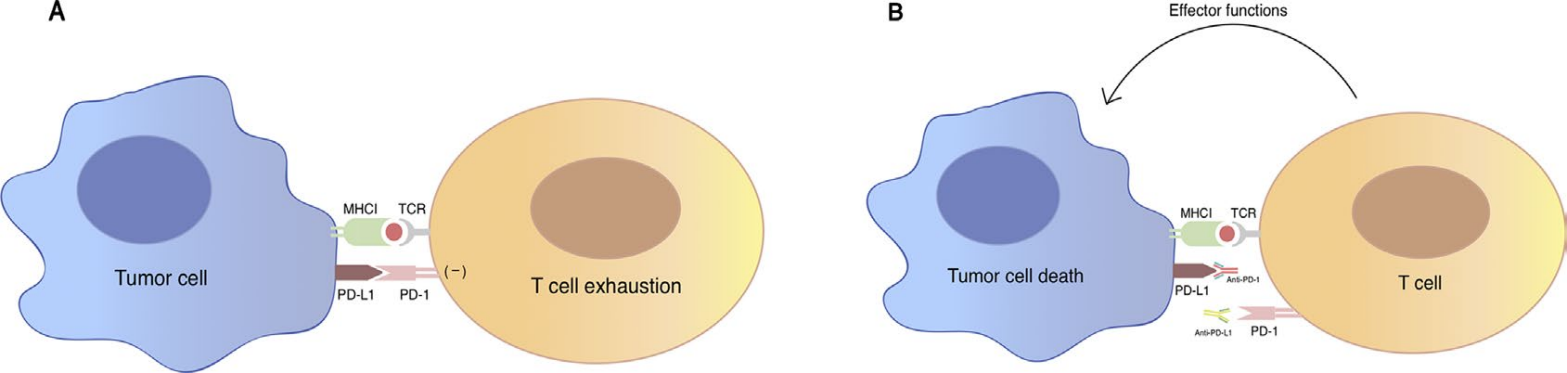
An illustration of PD-1/PD-L1 mediated immune tumor response. **(A)** PD-1 is a co-inhibitory molecule expressed on T cell, B cells, and myeloid cells. Binding of PD-1 to its B7 family of ligands PD-L1 on tumor cells results in suppression of proliferation and immune response of T cell, which are described as the “exhaustion” state of T cell. Activation of PD-1/PD-L1 signal pathway serves as a major mechanism of immune evasion by tumor cells. **(B)** Antibody blockade of PD-1 and PD-L1 reverses this process and enhances anti-tumor immune response. TCR, T-cell receptor; MHC, major histocompatibility complex; PD-1: programmed cell death protein 1; PD-L1: programmed death-ligand 1.

TCR (T-cell receptor)-mediated T cell activation is regulated by co-signaling molecules expressed on T cells, which can be divided into two classes: co-inhibitor and co-stimulator, based on their functional outcome. The balance between positive and negative adjustment of T cell activation relies on spatial and temporal expression of the co-stimulator and co-inhibitor ligands on tumor cells and antigen-presenting cells (Chen, 2004). PD-1 as an inhibitory checkpoint receptor is expressed on activated T cells, B cells, and myeloid cells. As a co-inhibitory molecule, PD-1 could lead to the attenuation of TCR-mediated signal after the engagement with its ligand PD-L1 (B7-H1) expressed on the surface of tumor cells or antigen-presenting cells in the tumor microenvironment (Freeman et al., 2000) (**Figure 1**). Recent studies suggest a novel mechanism that tumor cells might evade host immune attack through increased expression of PD-L1 (Dong et al., 2002). In tumor immune response, up-regulated PD-L1 molecule on tumor cell surface mediates T-cell anergy or exhaustion (Butte et al., 2007; Francisco et al., 2009). This up-regulation is possibly a result from pro-inflammatory cytokines such as interferon-γ produced by tumor infiltrating inflammatory cells (Dong et al., 2002).

Studies on murine models show the importance of PD-1/PD-L1 pathway in immune evasion in hematological malignancies and provide a rationale for targeting this pathway in clinical trial for leukemia patients. Scientists found that PD-L1 expression was up-regulated on C1498 (a murine AML cell line) when growing *in vivo*. PD-1 knockout mice could generate stronger immune response when transferred with C4198 and bore lower leukemia burden as well as showing longer survival. After using the antibody for PD-L1, similar results were obtained (Zhang et al., 2009). Another study on murine model found that co-expression of PD-1 and Tim-3 on CD8+ T cells increased during AML progression, and instead of blocking single pathway, combined PD-1/PD-L1 and Tim-3/galectin-9 blockade led to the reduction of tumor burden and lethality (Zhou et al., 2011). Treg cells play a negative part in anti-tumor immune response. In a systematic model of murine AML, tumor progression contributed to accumulation of regulatory T cells and elevation of expression of PD-1 molecules on CD8+ T cells in the tumor microenvironment. AML-associated Treg cells could suppress the function ability of activated CD8+ T cell. Using anti-PD-1 treatment on mice model prolonged the survival of CD8+ T cells at tumor sites, which led to tumor burden decrease and long-term survivors. Treg cell depletion following PD-1/PD-L1 blockade showed better therapeutic outcome. These data indicated a new approach of PD-1/PD-L1 blockade together with Treg cell depletion for treating AML patients by improving anti-tumor activation of AML-associated CD8+ T cell (Zhou et al., 2010).

Increasing data have shown a higher expression of PD-L1 in AML cells in some patients. And the expression level of PD-L1 was closely related to disease relapse, which was regarded as an independent negative prognostic factor (Chen et al., 2008). In order to illustrate the significance of checkpoint inhibitor expression level in tumor microenvironment, Daver and his partners performed 17-color multi-parameter flow-cytometry on bone marrow aspirates from 74 AML patients. Thirty-six of them were untreated AML patients and the rest were relapsed ones. This study showed that compared to healthy controls, PD-1 expression level was significantly higher in all T cell subpopulations both in untreated cohort (P < 0.05) and relapsed group (P < 0.006) (Daver et al., 2016). Other researchers found PD-1 expression level both on CD8+ and CD4+ T cell increased significantly at relapse stage after stem cell transplantation (Schnorfeil et al., 2015).

### Blockade of CTLA-4 in Acute Myeloid Leukemia

**Figure 2:**
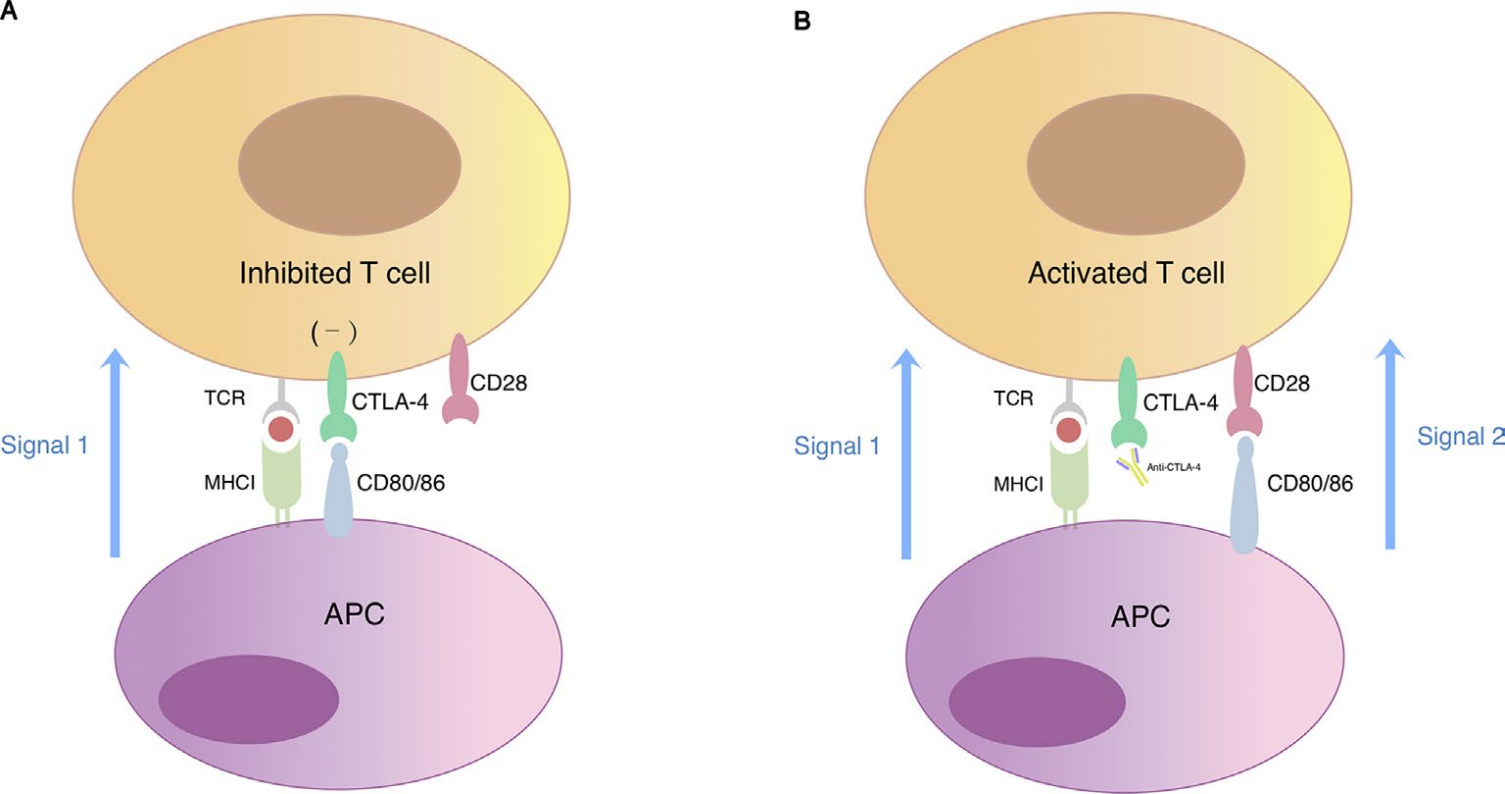
T cell activation regulated by CTLA-4 and CD28. **(A)** Simultaneous recognition of a specific major histocompatibility complex (MHC)–peptide complex by the T cell receptor (TCR) and of CD80/CD86 by the co-stimulatory receptor CD28 results in T cell activation. Cytotoxic T-lymphocyte-associated protein 4 (CTLA-4) is a CD28 homologue expressed on the surface of T lymphocytes with higher affinity for CD80/CD86. When CTLA-4 competitively binds to CD80/CD86, signal 2 required for T cell activation reduces, which eventually leads to T cell anergy. **(B)** The blockade of CTLA-4 signaling restores signal 2 in response to binding of CD28 with CD80/CD86 thus promoting T cell activation and proliferation.

CTLA-4 is a surface molecule expressed on activated T cells that regulates and mediates inhibitory signal to T cells. Sharing similar structure with its homologous T-cell co-stimulatory protein CD28 and with higher affinity to their common ligands, it competitively binds to CD80 and CD86 expressed by APCs thus resulting in negative effector T cell activation (**Figure 2**). CTLA-4 is an important mediator of self-tolerance and tolerance to tumor antigens. Treg cells often express high level of CTLA-4 and this could partly explain its suppressive function (Takahashi et al., 2000).

In an AML mouse model, persistent leukemic cells showed more resistance to specific cytotoxic T cells and presented higher expression level of PD-1 and CD80. Blocking of these PD-1 or CTLA-4/CD80 interaction could enhance CTL-mediated killing of persistent cells *in vitro* and prolonged mice survival *in vivo* (Saudemont and Quesnel, 2004). By analyzing AML patient samples, scientists found that 80% of AML samples tested at diagnosis constitutively expressed CTLA-4 and that CTLA-4 blockade might be a way to induce killing of leukemic cells through apoptosis (Pistillo et al., 2003; Laurent et al., 2007).

CTLA-4 blockade also plays a part in eliminating minimal residual disease (MRD) in AML. Dr. Saudemont found that when mice with residual disease were treated with anti-CTLA4 monoclonal antibody, persistent leukemic cells could be further cleared by enhanced CTL-mediated killing (Saudemont and Quesnel, 2004).

In a murine model, Dr. Blazar found that graft-versus-host effect was enhanced by anti-CTLA4 antibody infusion in the early course of post-bone marrow transplantation, which mainly depended on CD28. However, in the later course of post-transplantation stage, CTLA-4 blockade produced limited GVHD but augmented GVL effect of donor lymphocytes against host-derived leukemic cells (Blazar et al., 1999).

## Checkpoint Inhibition Therapy in the Clinic

### PD-1 Inhibition

The PD-1 inhibitors that are actively investigated in clinical trials include pidilizumab, nivolumab, pembrolizumab, durvalumab, and atezolizumab.

#### Nivolumab

Nivolumab is a human IgG4 anti-PD-1 monoclonal antibody. It is used as a first-line treatment for metastatic melanoma in combination with ipilimumab and as a second-line treatment for squamous non-small cell lung cancer as well as renal cell carcinoma (Johnson et al., 2015; Sundar et al., 2015). In 2016, the FDA approved nivolumab for patients with relapsed or progressed classical Hodgkin’s lymphoma after stem cell transplantation.

Aside from single agent approaches, scientists are trying to find novel therapeutic combinations of ICIs with other drugs to achieve better clinical outcome.

An interesting find is that epigenetic drugs could modulate the expression of checkpoint molecules on tumor-immersed lymphocytes as well as tumor cells. By treating MOLT-4 cells (a lymphatic leukemia cell line) with different concentration of 5-azacytidine, Zhang et al. found that PD-1 expression was positively related to the concentration of 5-azacytidine. This team demonstrated that PD-1 over-expression on lymphocytes was caused by the demethylation of promoter by 5-azacytidine, and changing the methylation state of PD-1 genes to recover T cell function could be a novel treatment direction (Zhang et al., 2011). Hypomethylating agent (HMA) 5-azacytidine was used as a standard regimen in treating older AML patients (Kantarjian et al., 2012). Yang et al. (2014) found that PD-1 as well as its two ligands PD-L1 and PD-L2 were up-regulated on CD34+ cells in patients with myeloid leukemia and their over-expression may contribute to treatment resistance to azacytidine. These evidences lead to several clinical trials combining epigenetic therapy with PD-1/PD-L1 blockade to improve response and survival rate in AML.

In an open-label, phase II study, Dr. Daver assessed the efficacy of combination therapy of nivolumab and azacytidine in R/R AML patients and the results were quite encouraging (Daver et al., 2019). This study enrolled 70 AML patients who previously received therapies including HMA. Among the 70 patients, the overall response rate was 33% including 16 (24%) patients who achieved complete remission (CR)/CR with incomplete blood count recovery (CRi)/partial remission (PR) and 7 of them reaching the standard of hematologic improvement. Six patients (9%) remained on study for over 6 months without either remission or clinical deterioration. The remaining 41 (58%) patients showed no response to therapy. Compared with historical controls in the entire population, the ORR of this study was higher with 33% versus 20%. In the subgroup of patients who did not receive HMA prior treatment, the superiority of new regimen was even more evident with ORR at 52% to 22%. The median overall survival (OS) was also higher in novel treatment group with 6.3 months versus 4.6 months (P = 0.013). Similarly, the event-free survival (EFS) was longer (4.2 vs 2.2 months). As for toxicities, grade 2 and grade 3–4 irAEs were observed in eight (11%) and eight (11%) patients respectively, which was similar to that observed in solid tumors. Among the patients with grade 2–4 side effects, pneumonitis was the most common with nine patients who suffered from such episodes. The rest included nephritis in six patients, skin rash related to immune response in three patients, and transaminitis in two. Steroids took effect on 88% of the patients who suffered from drug-related toxicities, and these 14 patients took on nivolumab treatment safely later on. Two patients died due to irAEs, both of which were refractory to steroids as well as subsequent infliximab therapy. Majority of the irAEs happened in the first 8 weeks after initial treatment of nivolumab. By performing multiparameter flow cytometry on bone marrow aspirates pre-therapy and on-therapy, they found that CD3+ and CD8+ T cells in the pre-therapy bone marrow aspirates were the best predictors of response, with the cut-off rate at 13.2% and 4.01%, respectively. These were well-recognized biomarkers in other solid tumors. CTLA-4 expression level on effector CD4+ and CD8+ T cells was increased in bone marrow aspirate samples from patients who showed no response to the treatment compared with responders. This indicated that the up-regulation of CTLA-4 was a potential mechanism of resistance to PD-1 blockade in non-responders, which had been seen in the therapeutic process in most solid tumors.

Another batch of enrolling cohorts conducted by the same team focused on frontline AML patients older than 65 years. In a 2017 ASH abstract, Daver et al. reported the preliminary results. Ten patients were treated with the combination of nivolumab and azacytidine with a median age of 75. Nine of them are evaluable for response: two CR, three CRp (CR with incomplete platelet recovery), one PR, one stable disease (SD) > 6 months, and two NR (no response) (Daver et al., 2017).

One year later, on the 60th ASH meeting, Dr. Daver reported their encouraging early findings on the study of treating salvage 1–2 R/R AML patients with nivolumab, azacytidine, and ipilimumab (NCT02397720) (Daver et al., 2018). Among the 14 evaluable patients, 43% of them achieved CR/CRi/CRp (n = 6). The median overall survival time for all patients was not reached and the projected 1-year overall survival rate was 58%.

On the same meeting, Dr Rita Assi and his colleagues reported their findings in a phase II study of accessing the addition of nivolumab to standard frontline therapy in patients with AML (NCT02464657) (Assi et al., 2018). This study enrolled 42 AML patients and 2 high-risk MDS patients with a median age of 54. Most of them were diagnosed with *de novo* AML (73%) and the remaining were therapy-related AML (7%) and high-risk myelodysplastic syndrome (4%). Nineteen patients had adverse genetic risk. Among the 44 evaluable patients, the ORR was 77% including 63% CR and 14% CRi. Thirty-four patients achieved CR or CRi, and among them, 18 patients were MRD (minimal residual disease) negative at the time of response. Nine of the remaining responders became MRD negative during additional follow-up at 1 to 3 months of nivolumab therapy. The median relapse free survival for patients who achieved response was 18.5 months and the median overall survival was 18.54 months. There was a trend of improved median OS when compared with a historical cohort of patients treated with cytarabine and idarubicin alone (mOS = 13.2 m). Concerning drug toxicities, the grade 3–4 adverse events were observed in six patients, including the rash found in two patients, colitis in two patients, and pancreatitis and transaminitis in one patient, respectively. Grade 3/4 cholecystitis in one patient possibly attributed to nivolumab. These events could be reversed by drugs. Eighteen patients proceeded to allo-SCT; 13 of them developed GVHD (grade I/II in 8, grade III/IV in 5). Eight patients with GVHD responded to treatment quite well. This group also performed multicolor flow cytometry studies and evidences showed that the co-expression of PD-1 and TIM3 (P = 0.04) on CD4-positive effector T cells in bone marrow was higher among non-responders compared with those who achieved remission, which indicated that up-regulation of TIM3 may contribute to drug resistance through some mechanism.

Using nivolumab in post-transplantation setting showed limited efficacy. Davids et al. (2018) reported severe adverse events in their phase I/Ib study on evaluating the safety of nivolumab in patients with relapsed hematological malignancies after allo-SCT. In the study, 28 patients were treated, with 11 relapsed AML patients. The median time post-transplantation was 21 months. Twenty-two patients were treated with 0.5 mg/kg nivolumab after two patients of first cohort (n = 6) on 1 mg/kg resulted in dose-limiting toxicity. However, accrual was terminated due to early GVHD and severe irAEs. Two patients developed grade III GVHD (liver and gut) together with grade 3 elevated bilirubin (n = 1) and grade 3 transaminitis (n = 1). Both of these two patients died from complications of GVHD. On the 0.5 mg/kg cohort, 10 patients (45%) had new onset or worsening GVHD. Other irAEs included grade 4 lipase elevation and grade 3 hypotension. Only one patient with AML achieved PR.

Eric et al. demonstrated the result of interim assessment on six patients with relapsed hematological malignancies treated with nivolumab after allo-SCT (Wong et al., 2018). Patients received 3 mg/kg nivolumab for up to 48 weeks. The median time from allo-SCT to first nivolumab administration was 25.5 months. Among the six patients, two AML patients showed no response with one participant achieving initial blast reduction (from 21% to 13%) but deteriorated in the end. Two patients developed grade III GVHD within the first 2 weeks after nivolumab treatment.

A number of trials evaluating nivolumab as a single agent in controlling AML and eliminating MRD are recruiting patients (NCT02275533, NCT02532231). **Table 1** lists currently active clinical trials of PD-1/PD-L1 inhibitors in AML.

**Table 1:** Ongoing clinical trials of immune checkpoint blockade in AML.

Setting	Target	Drug	NCT number	Phase of study	Study population	Therapy regimen	Objective	Status
AML and high-risk MDS	PD-1	Nivolumab	02464657	Phase I/II	AML or high-risk MDS	Nivolumab + idarubicin + cytarabine, single arm	MTD, EFS	Recruiting
Remission maintenance	PD-1	Nivolumab	02275533	Phase II	AML in remission	Single agent, two arms	PFS; OS, toxities	Recruiting
	PD-1	Nivolumab	02532231	Phase II	AML in remission, high risk for relapse	Single agent, single arm	RFS (time frame 6 months)	Recruiting
	PD-L1	Atezolizumab	03154827	Phase Ib/II	AML (>60 years) in remission	Atezolizumab + BL-8040	RFS (time frame: up to 5 years)	Recruiting
R/R AML	PD-1	Nivolumab	02397720	Phase II	R/R AML or elderly *de novo* AML patients	Azacitidine+nivolumab or azacitidine+nivolumab+ipilimumab, two arms	MTD, ORR of nivolumab with azacitidine, adverse event	Recruiting
	PD-1	Pembrolizumab	02768792	Phase II	R/R AML	Pembrolizumab after high-dose cytarabine as induction therapy	OR (CR/Cri); toxicities	Recruiting
	PD-1	Pembrolizumab	02845297	Phase II	R/R AML or elderly *de novo* AML patients	Pembrolizumab following azacitidine, single arm	MTD; ORR (CR, CRi)	Recruiting
	PD-1	Pembrolizumab	02996474	Phase I/II	R/R AML	Pembrolizumab and decitabine	Feasibility; efficacy	Active, not recruiting
	PD-1	Pembrolizumab	03291353	Early phase I	Refractory AML	Single agent, single arm	Adverse event; RR, OS	Recruiting
	PD-1/TIM-3	PDR001/MBG453	03066648	Phase I	R/R AML or *de novo* AML not suitable for standard therapy	Decitabine+PDR001 or decitabine+MBG453 or decitabine+PDR001+MBG453 or MBG453 alone or PDR001+MBG453	Safety, DLT	Recruiting
	CTLA-4	Ipilimumab	01757639	Phase I	R/R AML	Single agent, single arm	DLT, T-reg cell percentages; efficacy, PFS, OS	Completed
	CTLA-4	Ipilimumab	02890329	Phase I	R/R AML or elderly *de novo* AML	Ipilimumab and decitabine	MTD; clinical response	Recruiting
High risk or old age not eligible transplant	PD-1	Pembrolizumab	02708641	Phase II	Elderly AML (>60 years) not eligible for transplantation	Single agent, single arm	Time to relapse; OS	Recruiting
	PD-1	Pembrolizumab	02771197	Phase II	High-risk AML not eligible for transplant	Pembrolizumab following lymphodepletion therapy (fludarabine+melphalan), single arm	2-year relapse risk; safety	Recruiting
	PD-L1	Durvalumab	02775903	Phase II	Elderly AML (>=65 years) not eligible for transplantation	Durvalumab+azacitidine	ORR (CR/CRi)	Active, not recruiting
	CTLA-4	Ipilimumab	00039091	Phase I	AML in remission, not eligible for transplant	Single agent, single arm	Toxicities; ORR	Terminated
Post transplant	PD-1	Pembrolizumab	03286114	Phase I	AML relapse after allo-SCT	Single agent, single arm	Clinical benefit; response rate	Recruiting
	PD-1	Pembrolizumab	02981914	Early phase I	AML relapse after allo-SCT	Single agent, single arm	Adverse event; duration of response	Recruiting
	PD-1	Ipilimumab+ nivolumab	02846376	Phase I	AML after allo-SCT	Ipilimumab or nivolumab or ipilimumab+nivolumab	Safety (DLT); toxicities	Recruiting
	PD-1	Ipilimumab or nivolumab	01822509	Phase I/Ib	AML relapse after allo-SCT	Ipilimumab or nivolumumab, single arm	MTD, adverse events; RR, PFS, OS	Active, not recruiting
	PD-1	Ipilimumab or nivolumab	03600155	Phase I	High-risk AML or relapsed AML after allo-SCT	Nivolumab or ipilimumab or nivolumab+ipilimumab	MTD, DLT; ORR, DFS, OS	Recruiting
	CTLA-4	Ipilimumab	00060372	Phase I	AML after allo-SCT	Single agent, single arm	Safety dose	Completed
	CTLA-4	Ipilimumab	01919619	Early phase I	AML after allo/auto-SCT	Ipilimumab+lenalidomide	Toxicity rate (time frame: 28 days)	Recruiting

AML, acute myeloid leukemia; MDS, myelodysplastic syndrome; MTD, maximum tolerated dose; EFS, event-free survival; PFS, progression-free survival; OS, overall survival; RFS, recurrence-free survival; ORR, overall response rate; RR, response rate; DLT, dose limiting toxicity; DFS, disease-free survival; allo-SCT, allogeneic stem-cell transplantation; CR, complete remission; CRi, complete remission with incomplete hematologic recovery.

Objectives are listed as “primary; secondary” outcome measures.

#### Pembrolizumab

Another PD-1 blockade drug is pembrolizumab (formerly known as MK-3475 or lambrolizumab), an IgG4 isotype antibody. The FDA initially approved it in treating metastatic melanoma, and this drug was further approved to be used on unresectable or metastatic solid tumor with certain genetic anomalies (Syn et al., 2017).

Based on the previous study results, Dr. Joshua F. Zeidner conducted a multicenter phase II study to evaluate clinical outcome of the administration of pembrolizumab after high-dose cytarabine salvage chemotherapy (NCT02768792) (Zeidner et al., 2018). His group reported their early findings of this ongoing study on the 60th ASH meeting. Twenty-six R/R AML patients with median age of 54 had been evaluated for response and safety; 46% (n = 12) of the patients were in genetic adverse group according to ELN-risk standard. The overall response rate was 42% with nine CR/CRi (35%), one PR, and one MLFS (morphologic leukemia free state). Five of nine CR/CRi patients were MRD negative by standard monitoring. Four patients proceeded to allo-SCT in CR (n = 3) and MLFS (n = 1). Steroid responsive-grade II acute and moderate chronic GVHD was observed in two (50%) of them post-transplantation. With a median follow-up of 10.8 months, the median OS was 10.5 months. Most frequently observed grade 3 irAEs included hepatitis (n = 2), rash (n = 2), and epigastric pain of liver mass-lymphocytic infiltrate (n = 1). All the above events responded quite well to steroid treatment or resolved spontaneously without pharmaceutical intervention. Peripheral blood analysis revealed an increased diversity of TCR Vβ repertoire on CD8+ T cells in those who responded to PD-1 blockade therapy compared with non-responders. RNA-seq data from different cell fraction of bone marrow revealed specific gene expression profile correlated with response to therapy and these biomarkers were present prior to therapy.

Preliminary results of a single center, single arm trial of pembrolizumab (200 mg/m^2^) on day 1 in every 3-week cycle in R/R AML patients followed by decitabine (20 mg/m^2^) on days 8–12 and days 15–19 for 8 cycles were reported on the 60th ASH meeting (NCT02996474) (Lindblad et al., 2018). Ten patients with median age of 62 were enrolled, 7 with refractory disease and 3 with relapsed AML. Of the 10 evaluable patients, the ORR was 20% with one patient achieving MRD-negative CR and another one meeting the criteria of MLFS. With a median follow-up of 13 months to date, the mOS was 7 months. irAEs included grade 4 hypotension observed in one patient, grade 3 bilirubin elevation (n = 1), and diarrhea (n = 1). Two patients suffered from hypothyroidism (<grade 3) and another patient developed central diabetes insipidus that possibly attributed to pembrolizumab.

Pembrolizumab is also tested in post-transplantation setting in a prospective clinical trial. Justin Kline et al. (2018) reported an ongoing study of pembrolizumab for treatment of relapse of disease following allogeneic hematopoietic cell transplantation (NCT02981914). Eleven patients with hematopoietic malignancies including eight AML and three lymphoma were included. Out of these patients, seven were evaluable for response. AML patients showed modest response to pembrolizumab with two patients who had stable disease and another two who experienced disease progression. irAEs of any grade were observed in 63% of the patients. Grades 3–4 irAEs were reported in three patients with pneumonitis (n = 2) and hyperthyroidism (n = 1), which occurred within 3–6 weeks after pembrolizumab administration. These adverse events were resolved after pembrolizumab discontinuation and corticosteroid treatment.

#### Pidilizumab

CT-011 (Pidilizumab) is a humanized IgG1 monoclonal antibody that interacts with PD-1 to positively modulate antitumor immune response of T cells.

The interaction of this drug with PD-1 expressed on lymphocytes stimulates T cell activity and prolongs effector T cell survival. In a phase I clinical trial conducted by Berger et al., this drug was administered to patients with advanced stage hematological malignancies including eight AML patients, four of which had accepted allo-SCT previously. The result was rather modest with only one AML patient achieving a minimal response presented by a drop in peripheral blasts percentage from 50% to 5% at day 21 after administration of CT-011. This patient eventually had disease progression 61 weeks after initial treatment. No treatment-related toxicities were observed. The most frequent adverse event observed in the study is diarrhea, which occurred in one AML patient, but it may have resulted from GVHD instead of drug treatment. Another female patient received allo-SCT 8 weeks before enrollment, who was treated with the lowest dose of drug due to her early sign of GVHD. This patient eventually died from grade IV GVHD and persistent leukemia. It was unclear whether the deterioration of her disease was related to CT-011. Another three AML patients died due to serious adverse events, which were believed to be related with fulminated resistant leukemia (Berger et al., 2008). **Table 2** shows a summary of efficacy of ongoing clinical trials using checkpoint inhibitors in AML patients.

**Table 2:** Efficacy data of immune checkpoint inhibition in AML.

Agent	Pathway	Study design	Trial regimen	Study population	Response state	Overall survival	Comments
Pidilizumab	PD-1	Phase I	Single arm monotherapy	N = 8	Minimal response in 1 AML	NR	Limited efficacy as a single agent on AML, safe and tolerable dose as 0.2–6 mg/kg for advanced hematologic malignancies.
Nivolumab	PD-1	Phase II	Nivolumab+azacytidine in R/R AML	N = 70	ORR = 33% (CR/CRi = 15, PR = 1, HI = 7)	6.3m	Encouraging response rate and overall survival especially in salvage 1 (mOS = 10.6 months) and HMA naïve group (ORR = 52%)
Nivolumab	PD-1		Nivolumab+azacytidine in frontline elderly AML	N = 10	ORR = 60% (CR/CRp = 5, PR = 1)	NR	This trial is still enrolling
Nivolumab	PD-1	Phase II	Nivolumab, azacytidine, and ipilimumab on salvage 1–2 R/R AML	N = 14	ORR = 43% (CR/Cri/CRp)	NR	Projected 1 year os is encouraging at 58%. This trial is still enrolling.
Nivolumab	PD-1	Phase II	Nivolumab plus “3+7” standard therapy in AML	N = 42	ORR = 77% (CR = 28, Cri = 6)	18.5m	Addition to (I+A) induction is safe and feasible. Post-transplant severe GVHD is not significantly increased and is manageable.
Nivolumab	PD-1	PhaseI/Ib	Single arm in relapsed AML after allo-SCT	N = 11	PR in one AML patients	NR	Severe GVHD and irAEs occurred early and efficacy is modest.
Pembrolizumab	PD-1	Phase II	Pembrolizumab after HiDAC in R/R AML	N = 26	ORR = 42% (CR/CRi = 9, PR = 1, MLFS = 1)	10.5m	Pembrolizumab is well-tolerated in this setting. Response rate is encouraging without additive toxicities after HSCT.
Pembrolizumab	PD-1	PhaseI/II	Pembrolizumab followed by decitabine	N = 10	ORR = 20%	7 months	This first proof of principle study demonstrates the feasibility of the combination of pembrolizumab and decitabine in relapsed/refractory adult AML patients.
Pembrolizumab	PD-1		Pembrolizumab for relapsed AML after allo-SCT	N = 8	No patients showed response	NR	Treatment with pem in the post-alloSCT disease relapse setting is feasible, but can induce early and severe irAEs, for AML patients this regimen is less effective.
Ipilimumab	CTLA-4	PhaseI/Ib	Ipilimumab for R/R AML after allo-SCT	N = 12	ORR = 42%	With median follow up of 15 months, 12 month OS was 49%	CTLA-4 blockade was a feasible approach for the treatment of patients with relapsed hematologic cancer after transplantation. Complete remissions with some durability were observed, especially in extramedullary AML.

NR, not reported; ORR, over all response rate; OS, overall survival; CR, complete remission; CRi, complete remission with incomplete hematologic recovery; CRp, CR with incomplete platelet recovery; PR, partial response; HI, hematologic improvement; MLFS, morphological leukemia-free state; HMA, hypomethylating agents; HiDAC, high-dose cytarabine.

### CTLA-4 Inhibition

For patients with AML, allogeneic transplantation is a curative treatment option. Even so, there are still a portion of patients who would go through disease relapse after transplantation. The main mechanism for this therapy is contributed both by preparative regimen and more importantly by the immunologic GVL effect (Horowitz et al., 1990). Tumor cells escaping from the donor immune system contribute to relapse after allo-SCT. Based on evidences observed in murine model, CTLA-4 blockade to treat late relapse after transplantation by augmenting GVL effect seems a rational attempt.

Ipilimumab is a human IgG1 monoclonal antibody that antagonizes CTLA-4. It was first approved by the FDA for treating melanoma. This antibody has been explored in several solid tumors such as non-small cell lung cancer, small cell lung cancer, and bladder cancer.

The study evaluating ipilimumab on hematological malignancies conducted by Bashey enrolled 29 patients who underwent allo-SCT due to some certain malignancies but relapsed more than 90 days after last transplantation (Bashey et al., 2009). Patients were required to have not experienced grade III or IV acute GVHD and to be off immunosuppressive medications for more than 6 weeks before enrollment. They received ipilimumab as single infusion at dose between 0.1 and 3 mg/kg. Most of the patients in this cohort suffered from Hodgkin’s disease, and two AML patients were included. Median donor T cell chimerism on the day of ipilimumab infusion was 100%. Three patients demonstrated objective disease response but does not include any AML patients. Organ-specific immune irAEs were seen in four patients (14%) including grade 3 arthritis, grade 2 hyperthyroidism, and recurrent grade 4 pneumonitis. Dose-related grade 3 adverse events were anemia, thrombocytopenia, and neutropenia/fever, and grade 4 infection was observed. Most of grade 1 and 2 toxicities showed no clear relationship with the studied drug. No patient developed grade III or IV acute GVHD after ipilimumab alone. One AML patient treated at the dose level of 0.1 mg/kg developed grade 3 polyarthropathy clinically consistent with rheumatic arthritis and achieved complete regression of her symptom after being treated with corticosteroid.

In a phase I/Ib, open label, multicenter study of treating patients with relapsed hematological malignancies after allo-SCT with ipilimumab, 28 patients were enrolled who received two different dosages of ipilimumab (3 or 10 mg/kg) including 12 AML patients (Davids et al., 2016). The median time from transplantation to drug treatment was 22.5 months and median pretreatment T cell chimerism was 99%. Objective response was only observed in the cohort of patients who were treated on drug dose of 10 mg/kg with seven patients reaching the criteria for response. All responders had baseline donor T cell chimerism in the blood of 99% or higher, suggesting the important role of donor T cell in antitumor activity. Complete response was observed in five patients (23%), including three patients with leukemia cutis, one patient with myeloid sarcoma, and another one with AML developed from smoldering myelodysplastic syndrome with bone marrow involvement. With a median follow-up of 15 months, the 1-year survival rate was 49% and four patients who had a response continued to have a durable remission for more than 1 year. Toxicities were not specifically reported on AML cohort. On patients treated with 10 mg/kg ipilimumab, GVHD was observed in 3 out of 22 patients, including 2 cases of chronic GVHD of the liver and 1 case of grade II acute GVHD of the gut. All of these events were resolved with glucocorticoids but precluded further ipilimumab administration. Immune-related adverse events occurred in three patients including grade 2 immune thrombocytopenia, grade 3 colitis, and grade 2–4 pneumonitis, which responded to glucocorticoids. The incidences of grade 3 and 4 irAEs are listed in **Table 3**. One patient died of grade 3 colitis and grade 4 pneumonitis eventually. Exploratory studies were conducted to identify some possible predictors for response. Response was associated with *in situ* infiltration of CD8+ T cells as well as enrichment of effector T cell subsets.

**Table 3:** Immune-related adverse event rates associated with ICIs in acute leukemia.

	Nivolumab (0.5–3 mg/kg) (Daver et al., 2019; Davids et al., 2018; Assi et al., 2018)	Pembrolizumab (200 mg/m^2^) (Justin Kline et al., 2018; Lindblad et al., 2018; Zeidner et al., 2018)	Ipilimumab (0.1–10 mg/kg) (Bashey et al., 2009; Davids et al., 2016)
	≥Grade 3 (%)	≥Grade 3 (%)	≥Grade 3 (%)
Pneumonitis	1	18	3.4–4.5
Rash	4.5	7.6	
Pruritus	3		
Transaminitis	2–4		3.4
Colitis	1–4.5		4.5
Pancreatitis	2		
Elevated bilirubin	4	10	
Fatigue	1		
Hepatitis		7.6	
Hypotension		10	
Diarrhea		10	
Hyperthyroidism		9–14	
Arthritis			3.4

## Conclusions

Checkpoint inhibition treatment for AML is no doubt a major breakthrough. Preliminary data from ongoing clinical trials are promising especially for combination of PD-1 inhibitor nivolumab with HMAs with significantly higher response rate compared with historical control. In AML patients with extramedullary disease who relapsed post-transplantation, CTLA-4 inhibitor ipilimumab as a single agent shows a particular benefit. Due to the limited size of the early phase of clinical trials, more data are needed before we can better interpret these positive data and the response improvements observed in these trials need further validation. Despite the promising outcome from clinical trials, the introduction of checkpoint inhibitors is associated with unique irAEs, which are mostly reversible but can occasionally be fatal. Compared with toxicity resulting from conventional chemotherapy, immune-related irAEs caused by checkpoint inhibitors usually have a delayed onset and prolonged duration as well as a different toxicity profile (Fehrenbacher et al., 2016; Puzanov et al., 2017). Early recognition and proper intervention with immune suppression strategy, which is appropriate to affected organs, are key factors for effective management of irAEs. The areas of substantial interest for future study would be better innovative combinations to modulate immunologic targets and defining of biomarkers to select AML patients who are most likely to benefit from checkpoint inhibition therapy. Data from ongoing clinical trials emerging in the near future will guide further development of these agents while helping us gain understanding of how to minimize the risk of immune-related toxicities.

## Author Contributions

This study was DL’s original idea. DL also reviewed the literature and contributed to the manuscript writing and editing. TN mentored and contributed to the writing and editing of the manuscript. MW reviewed the literature and contributed to the manuscript writing and editing. YL contributed to the writing and editing of the manuscript. JL edited and proofread the manuscript.

## Conflict of Interest Statement

The authors declare that the research was conducted in the absence of any commercial or financial relationships that could be construed as a potential conflict of interest.

## Abbreviations

Allo-SCT, Allogeneic hematopoietic stem cell transplantation; AML, Acute myeloid leukemia; ASCT, Autologous stem cell transplant; CR, Complete remission; CRi, CR with incomplete count recovery; CRp, CR with incomplete platelet recovery; CTLA-4, Cytotoxic T-lymphocyte-associated protein 4; EFS, Event-free survival; GVHD, Graft-versus-host disease; GVL, Graft-versus-leukemia; HMA, Hypomethylating agents; ICIs, Immune checkpoint inhibitors; irAEs, Immune-related adverse events; MHC, Major histocompatibility complex; MRD, Minimal residual disease; ORR, Overall response rate; OS, Overall survival; PD-1, Programmed-death 1; PR, Partial remission; R/R, Relapsed/refractory; SD, Stable disease; TCR, T-cell receptor; Treg cells, Regulatory T cells.
